# Exact inter-discharge interval distribution of motor unit firing patterns with gamma model

**DOI:** 10.1007/s11517-018-01947-y

**Published:** 2019-01-26

**Authors:** Javier Navallas, Sonia Porta, Armando Malanda

**Affiliations:** Department of Electric, Electronic and Communication Engineering, Public University of Navarra, Pamplona, Navarra 31006 Spain

**Keywords:** Electromyography (EMG), Inter-discharge interval (IDI), Motor unit firing pattern, Motor unit potential train

## Abstract

Inter-discharge interval distribution modeling of the motor unit firing pattern plays an important role in electromyographic decomposition and the statistical analysis of firing patterns. When modeling firing patterns obtained from automatic procedures, false positives and false negatives can be taken into account to enhance performance in estimating firing pattern statistics. Available models of this type, however, are only approximate and use Gaussian distributions, which are not strictly suitable for modeling renewal point processes. In this paper, the theory of point processes is used to derive an exact solution to the distribution when a gamma distribution is used to model the physiological firing pattern. Besides being exact, the solution provides a way to model the skewness of the inter-discharge distribution, and this may make it possible to obtain a better fit with available experimental data. In order to demonstrate potential applications of the model, we use it to obtain a maximum likelihood estimator of firing pattern statistics. Our tests found this estimator to be reliable over a wide range of firing conditions, whether dealing with real or simulated firing patterns, the proposed solution had better agreement than other models.

**Graphical Abstract:**
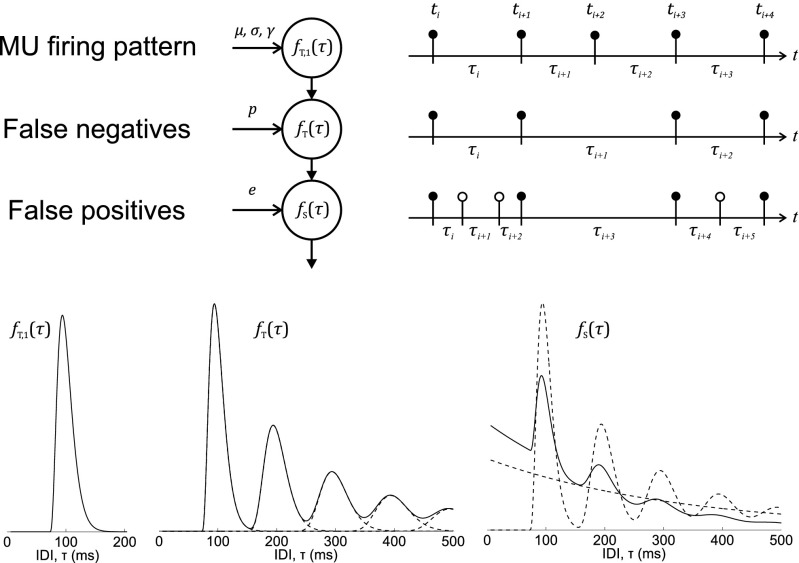
Model of the MU firing pattern generation and detection: *f*_*T*,1_(*τ*), IDI PDF of the physiological firing pattern; *f*_*T*_(*τ*), IDI PDF after modeling undetected firings (false negatives); *f*_*S*_(*τ*), IDI PDF after modeling classification errors (false positives)

Model of the MU firing pattern generation and detection: *f*_*T*,1_(*τ*), IDI PDF of the physiological firing pattern; *f*_*T*_(*τ*), IDI PDF after modeling undetected firings (false negatives); *f*_*S*_(*τ*), IDI PDF after modeling classification errors (false positives)

## Introduction

Analysis of the motor unit (MU) firing pattern provides invaluable information for EMG analysis [1–5] and the automation and evaluation of EMG decomposition [6–11]. The firing pattern under certain physiological conditions can be modeled as a renewal point process, and this approach has been demonstrated in physiological studies to be adequate [1–4, 7]. When a firing pattern is modeled as a renewal point process, the inter-discharge intervals (IDIs) are independent and equally distributed following a certain probability density function (PDF).

Two kinds of error may arise when the MU firing pattern is obtained from automatic decomposition methods [12–16]: false negatives, which are firings not detected in the decomposition process; false positives, which are firings not belonging to the MU but included in its firing pattern, i.e., classification errors. In the literature, there are models of IDI PDF that take into account false negatives [17, 18] and false positives [19]; all of these models assume a Gaussian model for the physiological IDI PDF.

Instead of a Gaussian distribution, a more suitable distribution for modeling the physiological IDI PDF is the gamma distribution [4]. This is so because the gamma distribution has nonnegative support, whereas the Gaussian distribution extends into negative values. Thus, models based on a Gaussian distribution can generate calculated IDIs that are negative, while measured IDIs can never be negative [20]. Additionally, the gamma distribution allows for some degree of skewness in the IDI PDF, which may enable models based on the gamma distribution to better reflect reality since physiological evidence indicates that the IDI PDF shows low-to-moderate skewness [2, 5, 21].

More importantly, the only published model that accommodates false positives [19] provides only an approximation of the IDI PDF. In the current paper, we show that, by applying statistical analysis to model the superposition of renewal point processes [22], it is possible to derive an exact solution of the IDI PDF that incorporates not just false positives but also false negatives.

The aim of the present study is to derive an exact IDI PDF model that takes into account false negatives and false positives, and in which a gamma distribution is used to model physiological IDIs. This paper presents the mathematical derivation of the model, and evaluates its usefulness in the context of estimation of MU firing pattern statistics.

## Methods

### Derivation of the model

#### Firing pattern and false negatives

**Fig. 1:**
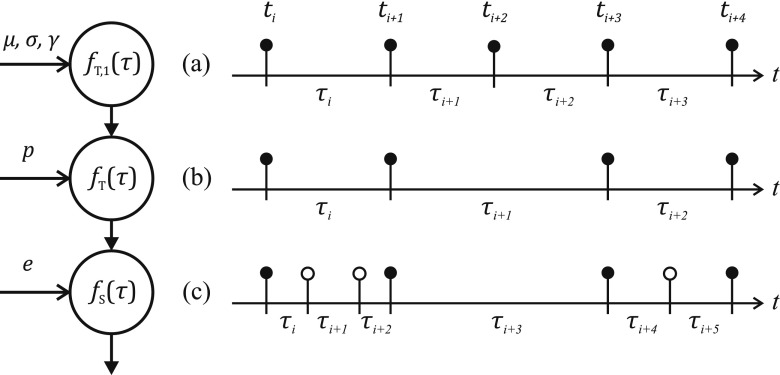
Schematic representation of the MU firing pattern point process with false negatives and false positives: **a** physiological MU firing pattern with firings in instants {*t*_*k*_} (black circles) and IDIs calculated as {*τ*_*k*_} = {*t*_*k*+ 1_ − *t*_*k*_}; **b** detected MU firing pattern where some of the firings are not detected, i.e., false negatives, leading to some IDIs being replaced by their summation; **c** detected MU firing pattern where some detection errors are included, i.e., false positives (white circles), leading to some physiological IDIs being split into two or more new IDIs

The MU firing pattern can be interpreted as a point process whose events take place in instants {*t*_*k*_} [1]. An MU firing pattern of short duration (< 10 s), under constant contraction conditions [3], and for which any possible MU synchronization is neglected, can be modeled as a renewal process [1]. In such a model, the physiological IDIs, calculated as {*τ*_*k*_} = {*t*_*k*+ 1_ − *t*_*k*_}, are independent and identically distributed following the same PDF, *f*_*T*,1_(*τ*) (Fig. 1a).

**Fig. 2:**
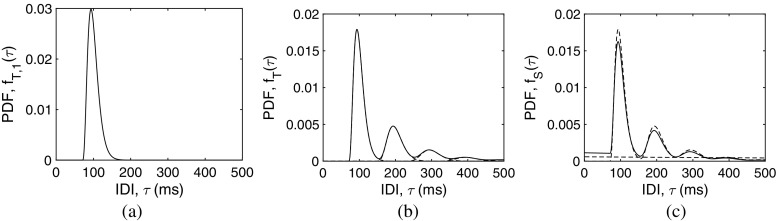
IDI PDFs of a MU firing pattern with parameters *μ* = 100, *σ* = 15, and *γ* = 1, and detection parameters *p* = 0.6, and *e* = 0.1: **a** IDI PDF of the original physiological MU firing pattern *f*_*T*,1_(*τ*); **b** IDI PDF of the thinned process *f*_*T*_(*τ*), after detection with probability *p*. Note that lost firings lead to side lobes of the PDF with means in *nμ*, and increasing standard deviation $\sqrt {n}\sigma $; (c) IDI PDF of the superposed process *f*_*S*_(*τ*), obtained by joining the thinned process *f*_*T*_(*τ*) and the Poisson process *f*_*E*_(*τ*) modeling false positives

A shifted gamma distribution, sometimes referred to as the Pearson type III distribution, of the IDI PDF is proposed in [4, 21] as a model that enables incorporation of a degree of skewness in the IDI PDF (Fig. 2a). The analytical expression is as follows:

1 $$\begin{array}{@{}rcl@{}} f_{T,1}(\tau) &=& \text{Gam}(\tau-\alpha;\rho,\beta) \\ &=& \left\{ \begin{array}{l} 0 \hfill , \tau \leq \alpha \\ \frac{(\tau-\alpha)^{\rho-1}}{{\Gamma}(\rho)\beta^{\rho}}\exp\left( \frac{\alpha-\tau}{\beta}\right) \hfill , \tau > \alpha \end{array} \right. \end{array} $$

where *α* is the location parameter, *β* is the scale parameter, and *ρ* is the shape parameter. These three parameters allow independent control of the physiological IDI mean *μ*, standard deviation *σ*, and skewness *γ*. The exact relationships between the parameters and the IDI PDF moments are [4] as follows:

2 $$ \alpha=\mu-\frac{2\sigma}{\gamma} ; \beta=\frac{\sigma\gamma}{2} ; \rho=\frac{4}{\gamma^{2}} . $$

To incorporate false negatives, the resulting point process is modeled as a thinned version [20] of the physiological MU firing pattern (Fig. 1b). In this way, individual firings can be regarded as being independently detected with probability *p*; hence, the probability that a firing go undetected is 1 − *p*. When a firing is not detected, the result will be a new IDI that is the summation of consecutive IDIs including undetected firings. The probability of not detecting *n* − 1 consecutive firings but detecting the *n* th equals *p*(1 − *p*)^*n*− 1^. Hence, the IDI PDF including false negatives can be expressed [4, 17, 18] as follows:

3 $$\begin{array}{@{}rcl@{}} f_{T}(\tau) = \sum\limits_{n = 1}^{\infty} p (1-p)^{n-1} f_{T,n}(\tau) \end{array} $$

where *f*_*T*,*n*_(*τ*) is the PDF of the IDIs obtained by summation of *n* consecutive IDIs.

For the gamma model, the summation of *n* gamma-distributed independent variables with equal *β* also follows a gamma distribution [23] that is calculated as follows:

4 $$\begin{array}{@{}rcl@{}} X_{i} &\sim& \text{Gam}(x_{i}; \rho_{i},\beta) \Rightarrow Y=\sum\limits_{i = 1}^{n} X_{i} \\ &\sim& \text{Gam}\left( y; \sum\limits_{i = 1}^{n} \rho_{i},\beta\right) \end{array} $$

where *x*_*i*_ = *τ* − *α*_*i*_ and $y=\tau -{\sum }_{i = 1}^{n} \alpha _{i}$. Given that all the IDIs are independent and identically distributed according to (1), the summation of *n* IDIs is distributed as follows:

5 $$\begin{array}{@{}rcl@{}} f_{T,n}(\tau) = \text{Gam}(\tau-n\alpha;n\rho,\beta) . \end{array} $$

Hence, Eq. 3 can be developed to obtain the IDI PDF of the thinned gamma process (Fig. 2a) as follows:

6 $$\begin{array}{@{}rcl@{}} f_{T}(\tau) = \sum\limits_{n = 1}^{\infty} p (1-p)^{n-1} \text{Gam}(\tau-n\alpha;n\rho,\beta) . \end{array} $$

Note that, according to Eq. 2, the mean IDI for each of the *f*_*T*,*n*_(*τ*) PDFs is as follows:

7 $$ \mu_{T,n}=n\mu=n(\alpha+\beta\rho) $$

while the mean IDI of the MU firing pattern process after incorporating false negatives is as follows:

8 $$ \mu_{T}=\frac{\mu}{p}=\frac{\alpha+\beta\rho}{p} . $$

#### Superposition of renewal processes

The possibility of false positives can be incorporated into the model by regarding the occurrence of false positives as a superposed point process [19]. The work of Cox and Smith [22] allows exact calculation of the PDF intervals between successive events of a superposition of renewal processes.

Given a renewal point process with intervals between successive events distributed according to *f*(*τ*), it is straightforward that

9 $$ F^{C}(\tau) = 1-F(\tau) = 1-{\int}_{0}^{\tau}\!\!\! f(t) \mathrm{d}t $$

where *F*(*τ*) is the cumulative distribution function and *F*^*C*^(*τ*) is the complementary cumulative distribution function or survival function.

The delay function is defined as the PDF of the intervals measured from a fixed time to the immediately preceding event [22]. The equilibrium delay function, *g*(*τ*), is the delay function a long time after the beginning of the process, and can be obtained [22] as follows:

10 $$ g(\tau)=\frac{F^{C}(\tau)}{\mu} $$

where *μ* is the mean IDI of the process. This equation can be reversed and combined with Eq. 9 to obtain [22] as follows:

11 $$ f(\tau)=-\mu\frac{\mathrm{d}g(\tau)}{\mathrm{d}\tau} . $$

We can define the complementary cumulative delay, *G*^*C*^(*τ*), as follows:

12 $$\begin{array}{@{}rcl@{}} G^{C}(\tau) = {\int}_{\tau}^{\infty}\!\!\!g(t) \mathrm{d}t = 1 - {\int}_{0}^{\tau}\!\!\frac{F^{C}(t)}{\mu}\mathrm{d}t . \end{array} $$

From this definition, we can elicit two relationships needed in later calculations as follows:

13 $$\begin{array}{@{}rcl@{}} \frac{\mathrm{d}G^{C}(\tau)}{\mathrm{d}\tau} = -\frac{F^{C}(\tau)}{\mu} \end{array} $$

14 $$\begin{array}{@{}rcl@{}} \frac{\mathrm{d}^{2}G^{C}(\tau)}{\mathrm{d}\tau^{2}} = \frac{f(\tau)}{\mu} \end{array} $$

The equilibrium delay functions are necessary because the superposition of *N* independent renewal point processes leads to a new renewal point process [22] where the complementary cummulative delay is as follows:

15 $$\begin{array}{@{}rcl@{}} G^{C}(\tau)=\prod\limits_{i = 1}^{N} {G_{i}^{C}} (\tau) . \end{array} $$

#### Firing pattern and false positives

If we neglect MU synchronization, firings from different MUs are independent. Hence, if a false positive is a random firing from a MU firing pattern other than that under analysis, the false positive error process can be modeled as a Poisson point process [19] with the following:

16 $$\begin{array}{@{}rcl@{}} f_{E}(\tau) = \lambda \exp(-\lambda \tau) \end{array} $$

17 $$\begin{array}{@{}rcl@{}} {F_{E}^{C}}(\tau) = \exp(-\lambda \tau) \end{array} $$

18 $$\begin{array}{@{}rcl@{}} {G_{E}^{C}}(\tau) = \exp(-\lambda \tau) \end{array} $$

where *λ* is the intensity of the false positive point process and can be calculated as follows:

19 $$\begin{array}{@{}rcl@{}} \lambda = \frac{1}{\mu_{E}} = \frac{ep}{\mu} \end{array} $$

where *μ*_*E*_ is the mean IDI between false positives and *e* is the ratio of false positive firings to true positive firings. The equality of ${F_{E}^{C}}(\tau )$ and ${G_{E}^{C}}(\tau )$ is due to the memorylessness of the Poisson point process [24].

When only two processes defined by PDFs *f*_*T*_(*τ*) and *f*_*E*_(*τ*) are superposed, reversing Eq. 14 and applying Eq. 15 leads to the IDI PDF of the superposed process (Fig. 1c) as follows:

20 $$\begin{array}{@{}rcl@{}} f_{S}(\tau) = \mu_{S} \frac{\mathrm{d}^{2} {G_{S}^{C}}(\tau)}{\mathrm{d}\tau^{2}} = \mu_{S} \frac{\mathrm{d}^{2}}{\mathrm{d}\tau^{2}} \left[G_{S}^{T}(\tau){G_{E}^{C}}(\tau)\right] \end{array} $$

where the mean IDI for the superposed process is as follows:

21 $$\begin{array}{@{}rcl@{}} \mu_{S} = \frac{\mu}{p (1+e)} . \end{array} $$

Deriving Eq. 20 gives the following:

22 $$\begin{array}{@{}rcl@{}} f_S(\tau) &=& \mu_S \left[ G_E^C(\tau) \frac{\mathrm{d}^2 G_T^C(\tau)}{\mathrm{d}\tau^2} + G_T^C(\tau) \frac{\mathrm{d}^2 G_E^C(\tau)}{\mathrm{d}\tau^2} \right.\\ &&\qquad\left. + 2 \frac{\mathrm{d} G_T^C(\tau)}{\mathrm{d}\tau} \frac{\mathrm{d} G_E^C(\tau)}{\mathrm{d}\tau} \right] \end{array} $$

and substituting Eqs. 13 and 14 into Eq. 22 leads to the following:

23 $$\begin{array}{@{}rcl@{}} f_{S}(\tau) &=& \frac{\mu_{S}}{\mu_{T}\mu_{E}}\left[\mu_{E} f_{T}(\tau) {G_{E}^{C}}(\tau) + \mu_{T} f_{E}(\tau) {G_{T}^{C}}(\tau)\right. \\ && \left. +\ 2 {F_{T}^{C}}(\tau){F_{E}^{C}}(\tau) \right] . \end{array} $$

Incorporating Eqs. 16, 17, and 18 into Eq. 23 enables calculation of the IDI PDF of any MU firing pattern, independently of its model, when superposed to a Poisson process modeling false positives. The resulting equation is as follows:

24 $$\begin{array}{@{}rcl@{}} f_{S}(\tau) = \frac{ep\exp(-\lambda\tau)}{\mu(1+e)} \left[\frac{\mu}{ep} f_{T}(\tau) + e {G_{T}^{C}}(\tau) + 2 {F_{T}^{C}}(\tau) \right] .\\ \end{array} $$

The survival function of the thinned process can be calculated as (Appendix A.3) follows:

25 $$\begin{array}{@{}rcl@{}} {F_{T}^{C}}(\tau) = \sum\limits_{n = 1}^{\infty} p (1-p)^{n-1} F_{T,n}^{C}(\tau) \end{array} $$

where (Appendix AA.2)

26 $$\begin{array}{@{}rcl@{}} F_{T,n}^{C}(\tau) = \left\{ \begin{array}{l} 1 \hfill , \tau \leq n\alpha \\ \frac{1}{{\Gamma}(n\rho)}{\Gamma}\left( n\rho,\frac{\tau-n\alpha}{\beta}\right) \hfill , \tau > n\alpha . \end{array} \right. \end{array} $$

The complementary cumulative delay of the thinned process can be calculated as (Appendix AA.3) follows:

27 $$\begin{array}{@{}rcl@{}} {G_{T}^{C}}(\tau) = \frac{1}{\mu_{T}}{\sum}_{n = 1}^{\infty} p (1-p)^{n-1} \mu_{T,n}G_{T,n}^{C}(\tau) \end{array} $$

where (Appendix AA.4)

28 $$\begin{array}{@{}rcl@{}} G_{T,n}^{C}(\tau) = \left\{ \begin{array}{l} 1-\frac{\tau}{n\mu} \hfill , \tau \leq n\alpha \\ \frac{1}{{\Gamma}(n\rho)} \left[ (n\alpha-\tau){\Gamma}\left( n\rho,\frac{\tau-n\alpha}{\beta}\right)\right.\\ + \left. \beta {\Gamma}\left( n\rho+ 1,\frac{\tau-n\alpha}{\beta}\right) \right] \hfill , \tau > n\alpha . \end{array} \right. \end{array} $$

Finally, Eqs. 25 and 27 make it possible to develop Eq. 24 so that we can obtain the exact PDF of the MU firing pattern with a gamma model taking into account both false negatives and false positives (Fig. 2c) as follows:

29 $$\begin{array}{@{}rcl@{}} f_{S}(\tau) = \frac{p}{\mu}\frac{e}{1 + e}\exp\left( \frac{-ep}{\mu}\tau\right) {\sum}_{n = 1}^{\infty} p (1 - p)^{n-1} K_{n}(\tau) \end{array} $$

where

30 $$\begin{array}{@{}rcl@{}} K_{n}(\tau) = \left\{ \begin{array}{l} 2+ep\left( n-\frac{\tau}{\mu}\right) \hfill , \tau \leq n\alpha\\ \frac{1}{{\Gamma}(n\rho)}\left[ \frac{\mu}{ep}\frac{(\tau-n\alpha)^{n\rho-1}}{\beta^{n\rho}}\exp\left( \frac{n\alpha-\tau}{\beta}\right)\right.\\ + \left. \left( 2+\frac{ep}{\mu}(n\alpha-\tau)\right){\Gamma}\left( n\rho,\frac{\tau-n\alpha}{\beta}\right)\right.\\ + \left. \frac{ep \beta}{\mu}{\Gamma}\left( n\rho+ 1,\frac{\tau-n\alpha}{\beta}\right) \right] \hfill , \tau > n\alpha . \end{array} \right. \end{array} $$

### Model-based maximum likelihood estimation

To test the usefulness of the IDI PDF model provided in Eqs. 29 and 30, a maximum likelihood estimator (MLE) of the model parameters was implemented. In essence, the MLE of the model parameters $(\hat {\mu },\hat {\sigma },\hat {\gamma },\hat {p},\hat {e})$ can be obtained for a given set of IDIs, $\{\tau _{k}\}_{k = 1}^{K}$, by applying optimization techniques [18, 19] to maximize the following:

31 $$\begin{array}{@{}rcl@{}} \mathcal{L}(\mu,\sigma,\gamma,p,e|\{\tau_{k}\}_{k = 1}^{K}) = \ln {\prod}_{k = 1}^{K} f_{S}(\tau_{k};\mu,\sigma,\gamma,p,e). \end{array} $$

The only limit that needs to be specified before being able to perform a numerical optimization of Eq. 31 is the truncation of the infinite summation in Eq. 29. Given that the *n* th term of the summation is weighted by *p*(1 − *p*)^*n*− 1^, the truncation error is related both to *N* (the upper limit of the truncated summation) and *p*. Even with a detection probability as low as *p* = 0.2, the weight of the 30th component, *K*_30_(*τ*), is just 3 ⋅ 10^− 4^, which, being 646 times smaller than the weight of the first component, is negligible in practical terms. Thus, by truncating the infinite summation after the 30th component (*N* = 30), precise numerical calculation of Eq. 29 is possible; smaller values of *N* may be chosen if it is deemed appropriate to trade precision for calculation speed.

In the current implementation, optimization was performed using Matlab’s *patternsearch* algorithm (MATLAB, The MathWorks, Inc., Natick, MA, USA) running on an Intel i7-2640M 2.8 GHz PC. Computation times for the MLE were always under 100 ms.

Parameter values chosen for the initial point from which optimization proceeds were similar to those used in other, previous estimation approaches (Table 1). $\hat {\mu }$ is initialized as the sample mode of the IDIs [18, 19, 25]; the most frequent value is expected to be close to the peak of the distribution, which is expected to be close to the real mean (Fig. 2c). $\hat {\sigma }$ is initialized as 0.2 times the sample mode of the IDIs [18, 19, 25], which leads to a coefficient of variation in the middle of the physiological range (from 0.1 to 0.3 [1]). $\hat {\gamma }$ is initialized to 0.2, in anticipation of a nonsymmetrical PDF with moderate skewness. $\hat {p}$ is initialized to 0.5 [18], a value in the lower range of the expected detection probability for automatically detected firing patterns [26, 27]. $\hat {e}$ is initialized to 0.05 [18, 19], which is a value in the middle of the range of the expected false positive rates for automatically detected firing patterns [26].

**Table 1:** Ranges and values for the IDI PDF parameters

Par.	Typical range	Optim. range	Initial guess	Sim. point
*μ*	30–160 ms^a^	$[0, \infty )$	mode(*τ*_*i*_)	100
*σ*	5–25 ms^a^	$[0, \infty )$	0.2 mode(*τ*_*i*_)	20
*γ*	− 0.50–2^b^	$[0.001, \infty )$	0.2	0.5
*p*	0.3–0.8	[0, 1]	0.5	0.7
*e*	< 0.1	[0, 1]	0.05	0.1

^a^ (see [1, 2]); ^b^ (see [5])

While in Eqs. 29 and 30, the solution is given in terms of *μ*, *α*, *β*, *ρ*, *p*, and *e*, a single set of parameters, such as (*μ*,*σ*,*γ*,*p*,*e*) or (*α*,*β*,*ρ*,*p*,*e*), can be used as the parameter-space for MLE optimization. If optimizing in the (*μ*,*σ*,*γ*,*p*,*e*)-space (hereafter, moment-space), the values of the IDI PDF parameters can be obtained by applying Eq. 2. Conversely, if optimizing in the (*α*,*β*,*ρ*,*p*,*e*)-space (hereafter, parameter-space), the IDI PDF moments can be obtained by applying as follows:

32 $$ \mu=\alpha+\beta\rho ; \sigma^{2}=\rho\beta^{2} ; \gamma^{2}= 4/\rho. $$

#### Log-likelihood curves

To evaluate any differences between the two optimization-space approaches, we carried a case-study simulation of the sensitivity of log-likelihood as a function of the model parameters. In this experiment, a total of 100 MU firing patterns were simulated with *μ* = 100, *σ* = 10, *γ* = 0.5, *p* = 0.7, and *e* = 0.05. For each simulation trial, a synthetic MU firing pattern was generated as a gamma renewal process with *μ*, *σ*, and *γ* and with enough discharges to fill 10 s. To model false negatives, each individual discharge had a probability 1 − *p* of being discarded. Additionally, false positives were modeled as extra firings, drawn at a rate *λ* = *ep*/*μ*, with a uniform distribution over the 10-s temporal span. Log-likelihood curves for each trial were calculated for a range of values for each parameter above and below the parameter’s real value while keeping the remaining parameters constant at their real value. In this way, the effect of IDI sample variability on log-likelihood maxima for each parameter was evaluated.

#### Simulated MU firing patterns

Estimation performance was tested with four simulation experiments. In all of these experiments, *μ* was kept fixed at 100 ms. In each run of each experiment, one of the other four parameters of the model, (*σ*,*γ*,*p*,*e*) was varied to take one of six different values, while the other three parameters were kept fixed at the simulation point values (Table 1). For each combination of the four parameters, 3 series of 1,000 trials were carried out. Firing patterns with false positives and negatives were simulated by means of the same procedure as that described in the previous section. Estimations of parameters were obtained by independently applying MLE with a Gaussian model [19] and a gamma model. Estimation with the gamma model was performed twice: once in the moment-space and once in the parameter-space. Each model was tested with one of the three independent simulation series. For each estimation result, the normalized error was calculated. Given the small number of IDIs in 10-s simulations, the actual values of *p* and *e* (that is, the values based on occurrence in the simulated patterns) were recalculated [19], after counting the false positives and false negatives of the simulated MU firing patterns, as *TP*/(*TP* + *FN*) and *FP*/*TP* respectively, where *TP* stands for the number of true positives, *FN* is the number of false negatives, and *FP* is the number of false positives.

The sample distributions of the estimates for each parameter combination were tested for bias by means of a one-sample *t* test (significance level: *α* = 0.01). The variances of the distributions were also tested with a one-sided *F* test for equal variance (significance level: *α* = 0.01), which indicates whether the variance of one distribution is significantly larger than that of another. This latter test was performed for the three MLE methods in pairs and in both senses (*A* bigger than *B* and *B* bigger than *A*). The results were then arranged to determine if the variance of a given method is significantly larger than that of just one of the other methods or both of them.

#### Real MU firing patterns

The algorithm was tested with 84 EMG signals of a 10-s duration recorded from the human *vastus lateralis* muscle with concentric needle electrodes during constant low-force isometric contractions. The study was approved by the Clinical Research Ethics Committee of Navarra. Informed consent was obtained from all subjects. The EMG signals were completely decomposed in the EMGLab environment [28]. The signal to interference ratio (SIR) [29] of the decomposed signals was calculated as a measure of the completeness of the decomposition. Only MU firing patterns with high-amplitude MUPs with a clearly distinguishable shape were accepted for the study. The decomposability index (DI) [15] of the decomposed MUPs was calculated as a measure of the MUP distinguishability. Nonstationary signals and signals without reliable and complete decomposition were discarded from the analysis, reducing the sample to 103 MU firing patterns.

Normality of the IDI sample of the MU firing patterns was tested with a Lilliefors test (significance level: *α* = 0.005); only 72 of the 103 MU firing patterns were found to be compatible with a Gaussian IDI distribution. The mean, standard deviation, and skewness of sample IDI statistics were calculated for each of the MU firing patterns.

Each of the 103 real MU firing patterns was corrupted by simulating a detection process of the individual firings with probability *p*, and adding false firings according to a uniform distribution over the 10-s span in a proportion *e*. The corruption process of each firing pattern was repeated independently five times for each combination of ten different values of *p* between 0.5 and 1, and ten different values of *e* between 0 and 0.5. In this way, we obtained 51500 corrupted MU firing patterns simulated to cover 100 different (*p*,*e*) combinations. Each of these trials was subjected to MLE estimation with a Gaussian model and to MLE estimation with a gamma model in the parameter-space in order to obtain estimates of $\hat {\mu }$ and $\hat {\sigma }$, and, with the gamma model, additionally $\hat {\gamma }$. In view of results obtained from the experiments described in the previous section (see Section 3.2), the moment-space version of gamma-based MLE method was not included in the analysis. The resulting parameter estimates were tested, with the Kolmogorov-Smirnov goodness-of-fit test (significance level: *α* = 0.05), to determine whether they were in agreement with the null hypothesis that the complete MU firing pattern conformed to a Gaussian distribution with the estimated parameters $\hat {\mu }$ and $\hat {\sigma }$, or to gamma distribution with the estimated parameters $\hat {\mu }$, $\hat {\sigma }$, and $\hat {\gamma }$.

The percentage of Kolmogorov-Smirnov tests in which the Gaussian and gamma model estimates were not rejected were calculated for each of the 100 (*p*,*e*) combinations. These percentages were calculated independently within the two MU firing pattern sets defined by the results of the Lilliefors test. To provide a comparison of the gamma and Gaussian models, we calculated the difference in the percentage of Kolmogorov-Smirnov rejections between both models.

## Results

### Log-likelihood curves

**Fig. 3:**
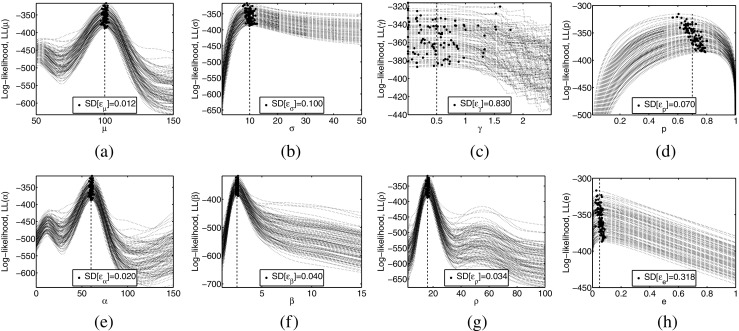
Log-likelihood curves (dotted lines) obtained for 100 realizations of a MU firing pattern with fixed parameters *μ* = 100, *σ* = 10, *γ* = 0.5, *p* = 0.7, and *e* = 0.05, when independently varying each parameter from the real solution (dashed lines). The maxima of the curves (points) correspond to the MLE for the parameter given the firing pattern realization. The curves are depicted as a function of: **a** the IDI mean *μ*; **b** the IDI standard deviation *σ*; **c** the IDI skewness *γ*; **d** the detection probability *p*; **e** the location parameter *α*; **f** the scale parameter *β*; **g** the shape parameter *ρ*; and **h** the false positive to true positive ratio *e*

The log-likelihood curves are depicted in Fig. 3. Curves fall into three groups: first, curves with clear maxima within the estimation range, such curves occurred for parameters such as *μ*, *α*, *β*, and *ρ*; second, curves with shallower variation within the estimation range, these curves were found for parameters such as *σ*, *p*, and *e*. Finally, curves that were almost flat, such as curves for *γ*. Directly related to shallowness in log-likelihood curves variation is the dispersion of the solutions (points in Fig. 3) around the real value, i.e., the standard deviation of the normalized estimation error (values in the legends in Fig. 3). This is the explanation of the higher spread of the results for some of the parameters: in decreasing order of standard deviation, *γ* (SD[*𝜖*_*γ*_] = 0.830), *e* (SD[*𝜖*_*e*_] = 0.318), *σ* (SD[*𝜖*_*σ*_] = 0.100), and *p* (SD[*𝜖*_*p*_] = 0.070). The results for the other four parameters had standard deviations below 0.040.

Although these results relate to estimation in a single point of the optimization-space, they suggest that the MLE optimization procedure performed better in the parameter-space than in the moment-space.

### Simulated MU firing patterns

**Fig. 4:**
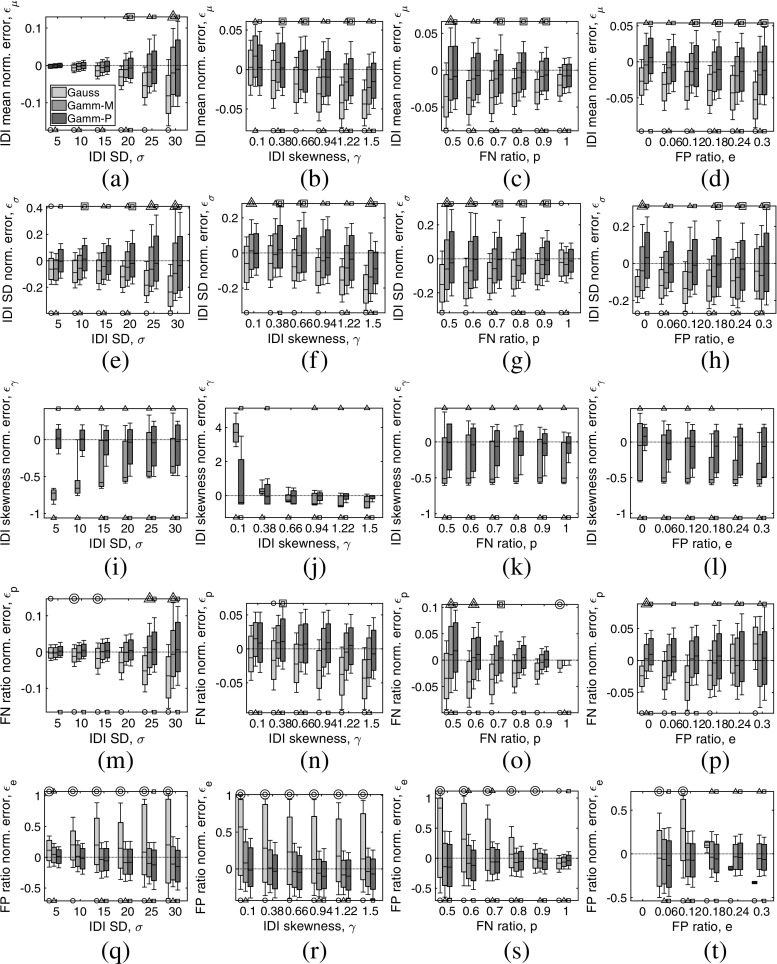
Normalized error of the (**a**-**d**) IDI mean, *𝜖*_*μ*_, (**e**-**h**) IDI SD, *𝜖*_*σ*_, (**i**-**l**) IDI skewness *𝜖*_*γ*_, (**m**-**p**) detection probability, (**q**-**t**) *𝜖*_*p*_, and false positive proportion, *𝜖*_*e*_, for the four simulation experiments: **a**, **e**, **i**, **m**, and **q** varying the IDI SD, *σ*; (**b**), (**f**), (**j**), (**n**), and (**r**) varying the IDI skewness *𝜖*_*γ*_; **c**, **g**, **k**, **o**, and **s** varying the detection probability, *p*; **d**, **h**, **l**, **p**, and **t** varying the false positive ratio, *e*. For each of the simulation experiments, the results of MLE estimation with the Gaussian model (light gray) are compared with those of MLE estimation with the gamma model in the moment-space, (*μ*,*σ*,*γ*), (medium gray) and with those of MLE estimation with the gamma model in the parameter-space, (*α*,*β*,*ρ*), (dark gray). In each case, the results are summarized with the median value and the 0.25 and 0.75 percentiles (grayed rectangles), and the 0.15 and 0.85 percentiles (whiskers) of the 1,000 simulations carried out for each parameter combination. Rejection of the hypothesis that there is no bias is marked with a small symbol under each of the distribution’s boxplots (circle, triangle, and square for Gaussian, gamma in moment-space, and gamma in parameter-space, respectively). Rejection of the hypothesis that the variance is not greater is marked with a small symbol (same ones as before) over each of the distribution’s boxplots, the symbol being doubled if the variance is significantly larger than that of the other two methods. The omission in the first set of results in **t** is only apparent and corresponds to the divergence of the normalized error when *e* = 0

Results of the experiments testing estimation performance are depicted in Fig. 4, with the estimated parameters arranged in rows and the varying parameters arranged in columns.

In terms of $\hat {\mu }$ (Fig. 4a–d), and $\hat {\sigma }$ (Fig. 4e–h), the Gaussian model presents bias in 45/48 cases, and tended to underestimate these parameters more severely with increasing *σ*, *γ*, and *e*, and decreasing *p*. The gamma model in the moment-space also presented bias, in 32/48 cases, although this bias is not so noticeable in the percentile plots. The gamma model in the parameter-space was only biased in 15/48 cases. In terms of variance, the Gaussian model had larger variance in 2/96 tests; the gamma model in the moment-space, in 44/96 tests; and the gamma model in the parameter-space in 64/96 tests. The three algorithms provided values of $\hat {\mu }$ within ± 5*%* of the real value, and values of $\hat {\sigma }$ within ± 15*%* of the real value.

Regarding $\hat {\gamma }$ (Fig. 4i–l), note that the Gaussian model does not provide an estimate of this parameter. The gamma model in the moment-space was biased in 24/24 cases, tending to overestimate the parameter. The gamma model in the parameter-space was biased in 19/24 cases, although this bias is not so noticeable in the percentile plots. The moment-space model had larger variance in 18/24 tests, while the parameter-space model had larger variance in only 3/24 tests. The two gamma models provided values of $\hat {\gamma }$ within ± 100*%* of the real value.

In the case of $\hat {p}$ (Fig. 4m–p), the gamma model in moment-space showed lower bias than the other models (22/24, 6/24, and 16/24 biased cases for the Gaussian, gamma in moment-space, and gamma in parameter-space, respectively). Although the Gaussian model tended to underestimate the parameter, for high values of *e*, it overestimated. In terms of variance, the Gaussian model had larger variance in 8/48 tests, the gamma model in the moment-space in 13/48 tests, and the gamma model in the parameter-space 14/48 tests. The three algorithms provided values of $\hat {p}$ within ± 5*%* of the real value.

For $\hat {e}$ (Fig. 4q–t), all the models were essentially biased (21/24, 15/24, and 17/24 biased cases for the Gaussian, gamma in moment-space, and gamma in parameter-space, respectively). In terms of variance, the gamma models were clearly superior to the Gaussian model (39/48, 6/48, and 6/48 cases of larger variance for the Gaussian, gamma in moment-space, and gamma in parameter-space, respectively). The three algorithms provided values of $\hat {e}$ within ± 50*%* of the real value, with the best results being obtained in low *σ* and high *p* conditions.

### Real MU firing patterns

**Fig. 5:**
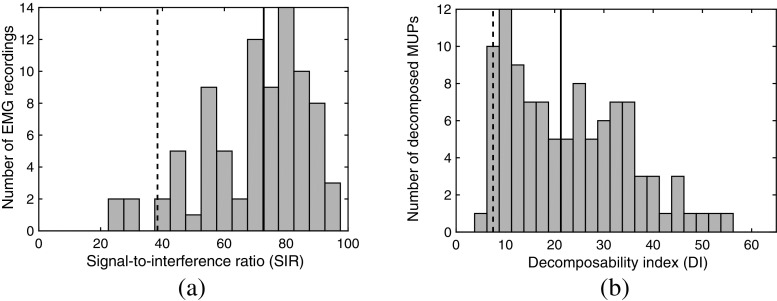
Evaluation of the supervised decomposition: **a** histogram of the SIR of the decomposed EMG signals; **b** histogram of the DI of the decomposed MUPs. In both histograms, the 5th percentile (dashed line) and the median value (solid line) of the sample are depicted

Histograms of the SIR of the decomposed EMG signals, and DI of the MUs are depicted in Fig. 5. The SIR is always greater than 27 (5th percentile is 38.4 and median value is 72.7) indicating the completeness of the decomposition. The DI is always greater than 5 (5th percentile is 7.5 and median value is 21.3) indicating the distinguishability of the MUPs from which the MU firing patterns are extracted [15].

**Fig. 6:**
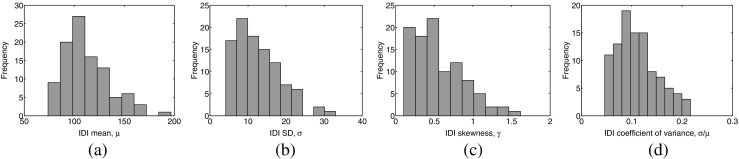
Histograms of the physiological IDI sample statistics of the 103 real MU firing patterns that were analyzed: **a** IDI mean, *μ*; **b** IDI standard deviation, *σ*; **c** IDI skewness, *γ*; and **d** IDI coefficient of variance, *σ*/*μ*. The observed values are in accordance with physiological studies, which report IDI means in the (30,160) ms interval [1, 2], IDI standard deviations in the (5,25) ms interval [1, 2], IDI coefficients of variance in the (0.1,0.33) interval [1], and IDI skewness in the (− 0.5,2) interval [5]

Histograms of the physiological IDI sample statistics of the 103 real MU firing patterns analyzed are depicted in Fig. 6. The observed values are in accordance with physiological studies reporting IDI means in the (30,160) ms interval [1, 2], IDI standard deviations in the (5,25) ms interval [1, 2], IDI coefficients of variance in the (0.1,0.33) interval [1], and IDI skewness in the (− 0.5,2) interval [5]. The Lilliefors test rejected a hypothesis of normality in 44 of the 103 complete MU firing patterns; in the remaining 59 patterns, normality was not rejected.

**Fig. 7:**
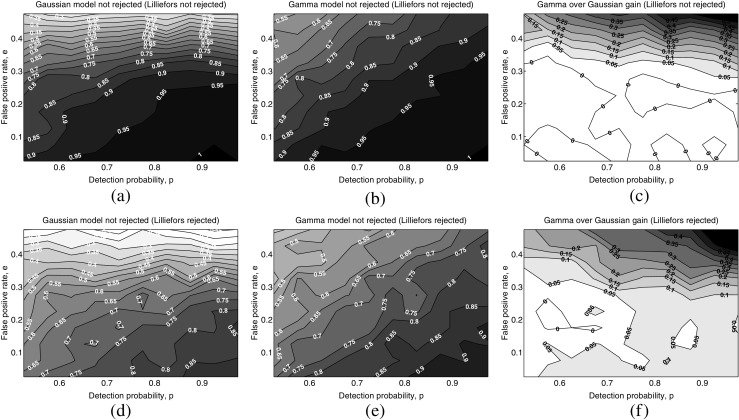
Summary of the Kolmogorov-Smirnov goodness-of-fit tests for the 103 real MU firing patterns when these patterns were corrupted with different combination values of *p* between 0.5 and 1 and *e* between 0 and 0.5, and with the MU firing patterns previously classified according to the results of the Lilliefors normality tests on the complete trains: **a** percentage of not-rejected estimations for the Gaussian model with the normality-not-rejected trains; **b** percentage of not-rejected estimations for the gamma model in the parameter-space with the normality-not-rejected trains; **c** difference of percentage of not-rejected estimations between the gamma model in the parameter-space and the Gaussian model with the normality-not-rejected trains; **d** percentage of not-rejected estimations for the Gaussian model with the normality-rejected trains; **e** percentage of not-rejected estimations for the gamma model in the parameter-space with the normality-rejected trains; **f** difference of percentage of not-rejected estimations between the gamma model in the parameter-space and the Gaussian model with the normality-rejected trains

Results of Kolmogorov-Smirnov tests of goodness-of-fit of the Gaussian and gamma models’ estimates are depicted in Fig. 7. The Gaussian model’s estimates fell into two distinct regions (Fig. 7a, d): first, when *e* < 0.3, the lower the *e* and the higher the *p*, the better the estimate; second, when *e* > 0.3, the estimate quality was almost independent of *p* and degradation of estimation was mainly a result of an increase in *e*. In the case of the gamma model (Fig. 7b, e), there was no differentiation of behavior as a function of *e*, and it was generally the case that the lower the *e* and the higher the *p* the better the estimate. These observations are corroborated by the figures for the differences in rejection percentages (Fig. 7c, f), which indicate that the main advantage of the gamma model over the Gaussian model in this respect appeared when *e* > 0.3 and this advantage increased as *e* increased.

For both models, there was a general loss in estimate quality when dealing with MU firing patterns whose normality had been rejected by the Lilliefors test. Quantitatively, normality-rejected firing patterns resulted in, on average, 15–20% more rejected estimates than did nonnormality-rejected firing patterns. With regard to the differences in estimate rejection percentages between models, the advantage of the gamma model over the Gaussian model was 5–10% greater when dealing with normality-rejected firing patterns than when dealing with nonnormality-rejected ones (Fig. 7c, d).

## Discussion

### Mathematical model of the MU firing pattern

The IDI PDF model that we developed in Section 2.1 and that assumes a gamma distribution for physiological IDIs has two important advantages over previous models: firstly, it is based on an exact analytical calculation, and this enables calculation of the PDF with unprecedented precision; secondly, it introduces a skewed distribution to model an IDI distribution. Several important features stem from each of these advantages.

The exactness of the model allows it to be used without restricting the values of its parameters. The validity of the earlier model [19] was restricted to conditions in which *e* < 0.2, that is, to circumstances in which false positives represented less than 17*%* of the overall IDIs. This limitation was due to the loss of precision for higher values of *e*. The existence of this shortcoming was corroborated in the present study; in experiments with both simulated and real signals, estimates with the Gaussian model worsened with increasing *e* (Fig. 4d, h, p, and t), and the goodness-of-fit plummeted as *e* increased beyond 0.3 (Fig. 7a, d). While this limitation may not be troublesome with some decomposed MU firing patterns of high quality [26, 27], it can be a severe problem with recordings taken under conditions of medium-to-high force muscle contraction, where it is not always possible to completely identify all the discharges due to superposition, and algorithms generally make more classification errors [10, 11]. As demonstrated in our experimental results, the current model, being exact, can be employed even in high *e* environments: estimates showed neither significantly more bias nor greater variance when *e* increased (Fig. 4d, h, p, and t), and the goodness-of-fit remained dependent on both *p* and *e*, even for *e* > 0.3 (Fig. 7b, e).

The use of a gamma model instead of a Gaussian model for the physiological MU firing pattern has two main benefits. On the one hand, the gamma distribution is strictly nonnegative and hence can be legitimately used as an inter-event distribution of a renewal point process in Eq. 1 [20]. The Gaussian distribution, because it allows inter-event values to be negative, can not be used unless suitably modified. Although the Gaussian distribution is commonly used because of the simplicity of the equations [4, 17–19], it is never formally correct.

In addition, use of a gamma distribution allows for a certain degree of skewness that may better reflect what is known about real MU firing patterns than a symmetric (zero skewness) distribution [4]. Although experimental evidence is not conclusive, different researchers have reported a small-to-moderate degree of skewness in the IDI distribution [2, 5, 21]. Whether a product of firing dynamics [5] or of unsteadiness in recording conditions [3, 4], variability needs to be accommodated, and accepting the possibility of a degree of skewness in the IDI distribution can help in this respect.

### Application to MU firing pattern estimation

In the set of real signals employed in the experiments reported here, a moderate degree of skewness was observed to be present (Fig. 6c) in MU firing patterns obtained in recording conditions that would traditionally be regarded as providing firing pattern stationarity [25]. Furthermore, over 40% of the MU firing patterns (44/103) were not compatible with the normality assumption in the Lilliefors test, and the Kolmogorov-Smirnov goodness-of-fit test consistently indicated that gamma model estimates had better fit than Gaussian model estimates (Fig. 7c, f).

With optimization procedures, special care must be taken to select the most appropriate parameter-space to evaluate (29). In our simulation experiments using the gamma model, results suggest that the parameter-driven MLE performed better than the moment-driven MLE in terms of the bias but worse in terms of the variance (Fig. 4). Summarizing, the gamma model in the moment-space was biased in 77/120 cases while the gamma model in the parameter-space was biased in 67/120 cases; significantly larger variance occurred in 81/216 cases and in 107/216 cases, respectively.

From the results obtained in the simulation experiments, we can ensure that, for the same amount of data available (length of the MU firing pattern), IDI PDF mean and standard deviation estimates obtained from the gamma model are less prone to bias than the estimates obtained from the Gaussian model at the cost of an increased variance. Hence, the relative amount of data to obtain reliable estimates of the gamma model parameters when applied to EMG decomposition should be of no relevance, given that it is has been shown to be superior to the Gaussian model-based estimates in 10 s recordings, which are typical in this kind of experiments. However, the stationarity requirement can be more problematic, given that IDI PDF asymmetry can arise from the nonstationarity of the MU firing pattern [4, 30]. Hence, careful assessment of MU firing pattern stationarity is always needed when trying to apply a stationary model to MU firing pattern parameter estimation [4].

### Further applicability of the model

Applicability of the IDI PDF model presented here is not limited to MU firing statistics estimation: the model can be useful in many statistically rigorous calculations concerning MU firing patterns, for example, in the development of EMG decomposition algorithms and in evaluation of EMG decomposition. Many automatic algorithms for EMG decomposition incorporate IDI PDF models [11, 13–15, 28], and IDI PDF statistics have been used to validate the MU firing patterns extracted by automatic EMG decomposition methods [27] and to derive a rigorous *a posteriori* calculation of EMG decomposition accuracy [7].

Besides being applied into new frameworks, new IDI PDF models can be easily implemented from Eq. 24 to accommodate other distributions for the physiological MU firing pattern, such as the Weibull distribution [30]. The resulting models will then incorporate modeling of both false negatives and false positives. In the same way, if a simpler, truncated-Gaussian model that overcomes the negative support of the distribution is developed, a Gaussian IDI can be implemented, reducing the physiological model parameters to *μ* and *σ*.

Although a Poisson distribution is a general and effective approach to model the false positive error process PDF [7, 19], it may not fulfill the criteria used during EMG signal decomposition. Hence, the false positive error process PDF could also be refined to fit the particular restrictions of a specific EMG decomposition algorithm, e.g., in [19], a correction to include a minimum allowable IDI value has been incorporated in terms of a truncation of the distribution.

## Conclusions

The presented IDI PDF model based on a gamma distribution is exact, is strictly nonnegative, and allows the introduction of a controlled degree of skewness into the physiological IDI distribution. Our test experiments demonstrate the feasibility of deriving an accurate MLE of physiological and detection parameters, and indicate that such a MLE can provide better results than previous models can.

## Appendix

### A.1 Calculation of ${F_{T}^{C}}(\tau )$

Calculation of the survival function of the thinned process is straightforward taking into account (6), as follows:

33 $$\begin{array}{@{}rcl@{}} {F_{T}^{C}}(\tau) &=& {\int}_{\tau}^{\infty}\!\!\! f_{T}(t)\mathrm{d}t = {\int}_{\tau}^{\infty}\!\!\! \sum\limits_{n = 1}^{\infty} p(1-p)^{n-1} f_{T,n}(t)\mathrm{d}t \\ &=& \sum\limits_{n = 1}^{\infty} p(1-p)^{n-1} {\int}_{\tau}^{\infty}\!\!\! f_{T,n}(t)\mathrm{d}t \\ &=& \sum\limits_{n = 1}^{\infty} p(1-p)^{n-1} F_{T,n}^{C}(\tau) \end{array} $$

### A.2 Calculation of $F_{T,n}^{C}(\tau )$

As *f*_*T*,*n*_(*τ*) is a piecewise-defined function, calculation of the corresponding survival function must also be done piecewise. Given Eq. 5 and applying Eq. 9, when *τ* ≤ *α*

34 $$\begin{array}{@{}rcl@{}} \left.F_{T,n}^{C}(\tau)\right|_{\tau\leq\alpha} = 1 - {\int}_{0}^{\tau}\!\!0 \mathrm{d}t = 1 \end{array} $$

when *τ* > *α*

35 $$\begin{array}{@{}rcl@{}} \left.F_{T,n}^{C}(\tau)\right|_{\tau>\alpha} &=& {\int}_{\tau}^{\infty}\!\!\! \frac{(\tau-n\alpha)^{n\rho-1}}{{\Gamma}(n\rho)\beta^{n\rho}} \exp\left( \frac{\tau-n\alpha}{\beta}\right)\mathrm{d}t \\ &=& \frac{1}{{\Gamma}(\rho)} {\Gamma}\left( n\rho,\frac{\tau-n\alpha}{\beta}\right) \end{array} $$

### A.3 Calculation of ${G_{T}^{C}}(\tau )$

Calculation of the complementary cumulative delay of the thinned process is straightforward taking into account (12) and (33), as follows:

36 $$\begin{array}{@{}rcl@{}} {G_{T}^{C}}(\tau) &=& {\int}_{\tau}^{\infty}\!\! \frac{{F_{T}^{C}}(t)}{\mu_{T}}\mathrm{d}t \\ &=& \frac{1}{\mu_{T}} {\int}_{\tau}^{\infty}\!\! \sum\limits_{n = 1}^{\infty} p (1-p)^{n-1} F_{T,n}^{C}(t)\mathrm{d}t \\ &=& \frac{1}{\mu_{T}} \sum\limits_{n = 1}^{\infty} p (1-p)^{n-1} \mu_{T,n} {\int}_{\tau}^{\infty}\!\! \frac{F_{T,n}^{C}(t)}{\mu_{T,n}}\mathrm{d}t \\ &=& \frac{1}{\mu_{T}} \sum\limits_{n = 1}^{\infty} p (1-p)^{n-1} \mu_{T,n} G_{T,n}^{C}(t)\mathrm{d}t \end{array} $$

### A.4 Calculation of $G_{T,n}^{C}(\tau )$

As $F_{T,n}^{C}(\tau )$ is a piecewise-defined function, calculation of the corresponding survival function must also be done piecewise. Given Eq. 26 and applying Eq. 12, when *τ* ≤ *α*

37 $$\begin{array}{@{}rcl@{}} \left.G_{T,n}^{C}(\tau)\right|_{\tau\leq\alpha} = 1 - {\int}_{0}^{\tau}\!\!\frac{1}{n\mu} \mathrm{d}t = 1-\frac{\tau}{n\mu} \end{array} $$

On the other hand, when *τ* > *α*

38 $$\begin{array}{@{}rcl@{}} \left.G_{T,n}^{C}(\tau)\right|_{\tau>\alpha} &=& {\int}_{\tau}^{\infty}\!\!\! \frac{1}{n\mu{\Gamma}(\rho)} {\Gamma}\left( n\rho,\frac{\tau-n\alpha}{\beta}\right)\mathrm{d}t \\ &=& \frac{1}{n\mu{\Gamma}(n\rho)} \left[ (n\alpha-\tau){\Gamma}\left( n\rho,\frac{\tau-n\alpha}{\beta}\right)\right. \\ && + \left.\beta {\Gamma}\left( n\rho+ 1,\frac{\tau-n\alpha}{\beta}\right) \right] \end{array} $$
